# A Qualitative Study of the Cognitive Rehabilitation Program GRADIOR for People with Cognitive Impairment: Outcomes of the Focus Group Methodology

**DOI:** 10.3390/jcm10040859

**Published:** 2021-02-19

**Authors:** Eider Irazoki, Mª Cruz Sánchez-Gómez, Leslie María Contreras-Somoza, José Miguel Toribio-Guzmán, Mª Victoria Martín-Cilleros, Sonia Verdugo-Castro, Cristina Jenaro-Río, Manuel A. Franco-Martín

**Affiliations:** 1 Department of Personality, Evaluation and Psychological Treatments, Faculty of Psychology, Campus Ciudad Jardín, University of Salamanca, Avenida de la Merced, 109, 37005 Salamanca, Spain; lesliecontreras@usal.es (L.M.C.-S.); crisje@usal.es (C.J.-R.); mfm@intras.es (M.A.F.-M.); 2 Department of Research and Development, INTRAS Foundation, Carretera de la Hiniesta 137, 49024 Zamora, Spain; jmtg@intras.es; 3 Department of Didactic, Organization and Research Method, University of Salamanca, Paseo Canalejas, 169, 37008 Salamanca, Spain; mcsago@usal.es (M.C.S.-G.); viquimc@usal.es (M.V.M.-C.); soniavercas@usal.es (S.V.-C.); 4 Department of Psychiatry, Rio Hortega University Hospital, Calle Dulzaina, 2, 47012 Valladolid, Spain; 5 Department of Psychiatry, Zamora Hospital, Calle Hernán Cortés, 40, 49071 Zamora, Spain

**Keywords:** cognitive rehabilitation, computer-based intervention, psychosocial intervention, cognitive impairment, dementia, focus group

## Abstract

In recent years, technology has been implemented in the field of interventions for older adults. GRADIOR 4.5 is a cognitive software within the wide variety of available multimedia programs that support healthcare professionals in cognitive assessment and neuropsychological rehabilitation. The study aimed to evaluate the new version of GRADIOR (v4.5) based on the experience of people with mild cognitive impairment (MCI), people with dementia (PWD), and healthcare professionals. A qualitative study using the focus group methodology was carried out involving 13 people with MCI, 13 PWD, and 11 healthcare professionals. An analysis of the content and the level of feedback was performed. The study showed that GRADIOR 4.5 might be sufficiently adapted to PWD and people with MCI. Participants were motivated to use GRADIOR 4.5, showed high acceptability of the software, and a positive attitude towards technology. However, healthcare professionals suggested significant improvements to the software. GRADIOR 4.5 appeared to be a promising intervention that, because of its positive experience and acceptability, could be systematically implemented to complement cognitive rehabilitation interventions for older adults with MCI and dementia. Finally, it is advisable to consider the suggestions gathered in this study for future developments.

## 1. Introduction

As a result of better living conditions, the number of older adults has substantially increased. One of the main problems of aging is cognitive and functional impairment impacting the quality of life. As pharmacological treatments have been found to have modest efficacy for these problems [1], the effort to use non-pharmacological interventions has doubled in recent decades [2]. The primary aim of this type of intervention is to reduce cognitive impairment progression, enhance social interactions and daily activities, and support caregivers [3].

Computer-based cognitive interventions are considered a prominent therapeutic tool in the field of neurocognitive disorders’ treatment. Undoubtedly, the primary benefit of using technology as part of cognitive therapies is that interventions are more accessible, flexible [4], and cost-effective [5]. Furthermore, using portable devices such as tablets and computers enables bringing interventions to rural areas or users’ homes [6]. Moreover, computer-based interventions support clinical practice and considerably reduce therapists’ burden [7]. However, there are also disadvantages and limitations related to computer-based interventions. Interventions using technology are commonly delivered individually and often with no supervision. Therefore, there is less contact with therapists and a lack of monitoring of emotional aspects such as fatigue or frustration. Another disadvantage could be the need to use accessories such as a keyboard and mouse, which require more significant cognitive effort and coordination [8]. Learning difficulties have also to be overcome, training older adults to use existing technologies or developing new products tailored to their requirements [9,10]. It is also noteworthy that technology is not always available in rural areas.

Despite the increased use of technology by older people, electronic equipment is still associated with younger generations. People in the elderly population have been excluded from the use of diverse technologies due to impaired cognitive ability and a lack of experience [11]. Moreover, older people may feel uncomfortable when faced with new technologies because of a lack of education [12]. Fortunately, the perception and attitude of older people towards digital devices have changed profoundly, and computers and tablets are now perceived as potential tools to improve their health and wellbeing [13].

Unlike in the past, it is now considered vital to ask people with dementia about their experience and needs to understand what requirements should be addressed [14]. The most important thing is to develop suitable technologies to ensure success [15], but without ignoring the importance of all target groups’ participation. Involving people with dementia in the different phases of technology development could be a reliable strategy to achieve these goals [14,16]. The purpose of involving people with dementia in the processes concerning them contributes to social inclusion and the quality of care provided [17]. Professionals’ (e.g., psychologist, social workers) appraisal is also needed. Professionals can highlight the benefits and positive effects of using technology and emphasize the difficulties and challenges concerning technology implementation.

The cognitive rehabilitation program GRADIOR 4.5 (INTRAS Foundation, Valladolid, Spain) is a multimedia software designed to support professionals in cognitive assessment and neuropsychological rehabilitation [18]. The software was specially designed for different types of cognitive impairment or disabilities (e.g., dementia, mental illness) and contains more than 12,500 cognitive exercises in diverse modalities (e.g., memory, orientation, attention) and sub-modalities (e.g., verbal memory). Furthermore, GRADIOR 4.5 has a user-friendly appearance and an intuitive interface improved by different studies focused on enhancing usability and user experience [19,20]. The current design facilitates direct interaction through a touchscreen device. It is not necessary to be an experienced user of modern technology to work with the device, since the program supports users with visual and auditory instructions until tasks are completed.

GRADIOR includes an independent section for the therapist that enables creating personalized rehabilitation treatment sessions, monitoring cognitive exercises, and accessing execution reports after each treatment session. The program was designed for autonomous use; however, it is convenient for therapists to supervise the sessions and support users [18].

Currently, the GRADIOR program is available for healthcare providers, and it is distributed through its supplier, INTRAS Foundation [21]. The software has been used for several years now, but it is still under constant updates and modifications. Recently, a new version of the program has come out. The recent version incorporates new functions, new types of exercises, and usability and design improvements. GRADIOR changed its general appearance by renewing its image and including a new logo compared to previous versions. In most cognitive exercises, real images were added instead of drawings, turning the tasks into being more ecological, familiar, and nearer to the subject’s reality. Furthermore, changes were made to the therapist’s management system, trying to optimize it and making it even easier to use [9].

Usability and acceptability studies are essential for developing suitable technologies since effective but non-applicable software is useless [22,23]. The program’s effectiveness was analyzed in healthy older adults and people with mild impairment combined with a physical program within the Long Lasting Memories Project [24]. Furthermore, a usability study with Spanish older adults was carried out [19]. Recently, GRADIOR was analyzed through a questionnaire in order to establish the degree of satisfaction and usability in people with different clinical conditions [25,26]. However, this study is the first to evaluate GRADIOR through the focus group methodology. This research was part of a usability study of a clinical trial currently being conducted to establish the effectiveness of GRADIOR for neurocognitive rehabilitation in people with mild dementia and mild cognitive impairment [27]. The study aimed to evaluate the latest version of GRADIOR (v4.5) based on the experience and opinions of potential users, such as people with mild cognitive impairment (MCI), people with dementia (PWD), and healthcare professionals. The study’s findings will be relevant for fitting the software to the users’ unmet needs and preferences, improving usability, and promoting its implementation.

## 2. Materials and Methods

### 2.1. Design

A qualitative study using the focus group methodology was carried out. This qualitative method creates favorable environmental conditions for spontaneous expression and interaction between the participants, encouraging people to share ideas, beliefs, experiences, or opinions during guided discussions. This study followed the COREQ (COnsolidated criteria for REporting Qualitative research) checklist for qualitative research [28].

### 2.2. Study Participants

A total of 37 people agreed to participate in the study. Six focus group sessions were conducted: two focus groups for people with MCI, two focus groups with PWD, and two focus groups with healthcare professionals (PR). Table 1 shows the sociodemographic characteristic of all participants.

All participants were selected by convenience sampling. Potential participants to be included in the dementia and MCI groups were contacted by telephone. The study purpose was explained in detail to all participants, including voluntary participation, personal data protection, and estimated time for the meetings. Additionally, the usual caregivers of each PWD were contacted in order to consider the involvement of their relatives in the study. Relevant information was also provided to caregivers regarding the study aims and confidentiality of the data. Health professionals were contacted by email. They were also informed before the study about the objectives and methodology of the meetings.

#### 2.2.1. MCI Group

The recruitment of people with MCI was conducted from the memory workshop provided by INTRAS Foundation in Zamora. The criteria used to define MCI participants were the same as those established for the clinical trial to which the study corresponds [27]. Accordingly, the psychiatrist of the research team verified that participants with MCI complied with the criteria of Petersen [29,30]. As an exception, a middle-aged adult participant with early cognitive impairment was included. Furthermore, participants had previous experience with GRADIOR 4.5 and fulfilled the memory workshop requirements: (i) aged over 55 years; (ii) preserved vision and hearing; (iii) basic writing and reading level; (iv) subjective memory complaints; and (v) cognitive impairment. The criterion for cognitive impairment was defined by a cut-off point of ≤27 for the Mini-Mental State Examination (MMSE) score [31].

Thirteen people with MCI participated in the study (age 75.31, SD = 8.04). Most participants were women (84.61%), had primary education (53.85%), were widowed (53.8%), and had 2–3 years of experience with GRADIOR 4.5 (46.2%). Participants were divided into two sub-groups (G1 = 5; G2 = 8) to facilitate sharing ideas and opinions.

#### 2.2.2. PWD Group

PWD was recruited from the memory workshops and memory clinic of INTRAS Foundation in Zamora. The criteria for defining dementia were also the same as in the study protocol [27]. Participants with dementia met the Diagnostic and Statistical Manual for Mental Disorders (DSM-5) diagnostic criteria for major neurocognitive. This group included a middle-aged adult with early dementia. The inclusion criteria for this group were: (i) experience with the new version of GRADIOR (v4.5); and (ii) clinical diagnosis of mild dementia. All participants of this group had previous cognitive assessments, so the research team’s psychiatrist reviewed all the cases. The MMSE cut-off point for people with dementia was <25 points [32].

Thirteen PWD participated in the study (age 76.64, SD = 10.14). Most participants were women (69.2%), had primary education (46.2%), were widowed (53.8%), and had less than one year of experience with GRADIOR 4.5 (61.53%). Participants were also divided into two sub-groups (G1 = 6; G2 = 7).

#### 2.2.3. Healthcare Professionals’ Group

Healthcare professionals participating in the focus groups were INTRAS Foundation employees. The inclusion criteria for professionals were (i) working or having worked as intervention professionals with PWD (e.g., psychologist, social workers, occupational therapist) and (ii) experience with GRADIOR 4.5 or any other similar cognitive training software. Healthcare professionals were not involved in the design and development of the GRADIOR program. They were working on clinical interventions with people with cognitive impairments at the time of the study.

Eleven healthcare professionals participated in the focus group sessions (age 33.09, SD = 7.91). Most participants were women (72.7%) and had less than one year of previous experience with the last version of GRADIOR (45.5%). Professionals were also divided into two sub-groups (G1 = 6; G2 = 5).

### 2.3. Data Collection

The study took place in a memory clinic in Zamora (Spain). The data collection was carried out between July 2017 and January 2018. Only the first and third authors were present during the focus groups. Both research members were females, neuropsychologists, and Ph.D. students in contact with older adults and received previous training in the focus group methodology. The first author moderated all the focus groups.

All the focus groups were conducted in a quiet environment and following the same guidelines. At the beginning of each discussion, the moderator explained the study aim and asked the participant to talk freely. Emphasis was placed on the audio and video recording and data confidentiality. All participants agreed to sign the written informed consent and filled in the sociodemographic information. The discussions were guided through semi-structured open-ended question scripts (Table 2). The text was the same for the MCI and PWD groups, and it included aspects such as the user interface, device characteristics, and program usefulness. The script for professionals discussed extra content, such as the therapist’s profile characteristics. Healthcare professionals were asked to evaluate GRADIOR 4.5 as part of a cognitive intervention for people with MCI and dementia and as a support tool for their professional work.

Before each topic, the moderator provided short descriptions of each question to facilitate the discussion, especially in the PWD and MCI groups. During the discussions, the moderator encouraged participants several times to report at least one aspect of the program to be improved. Thus, it was ensured that negative aspects were also discussed. On average, the meetings lasted 39 min for the MCI group, 36 min for the PWD group, and 57 min for the healthcare professionals’ group.

### 2.4. Data Analysis

**Figure 1:**
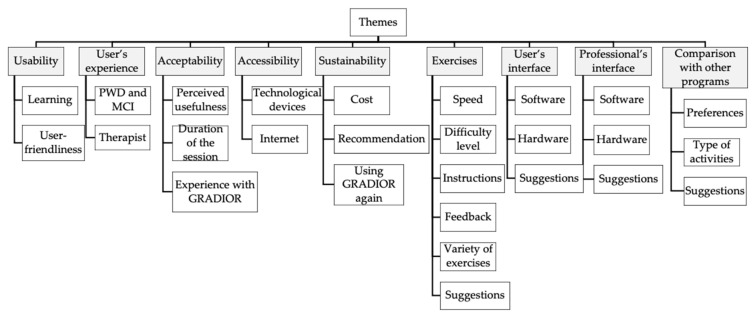
Relevant themes for the study purpose.

The focus group assistant transcribed all the discussions verbatim and anonymously. The data analysis was carried out with the Nvivo 12 Plus program (QSR International, Melbourne, Australia), which facilitates coding and managing nodes. The content analysis and data coding were performed by independent researchers from the University of Salamanca. Relevant themes for the study were previously identified, and new ones were also generated. The final topics discussed were: (a) usability; (b) user’s experience; (c) acceptability; (d) accessibility; (e) sustainability; (f) exercises; (g) user’s profile; (h) therapist’s profile; and (i) comparison with other programs (Figure 1). For obvious reasons, the therapists’ profile was only commented on in the group of healthcare professionals.

The sociodemographic information of study participants was analyzed first (Table 1). Secondly, a homogeneous system of categories was designed, and a double-entry matrix system was used to examine the results (participant X category). Thus, the content was analyzed within the same codification system, and the results were compared by category and participant group. Table 3 summarizes the ideas expressed by the study participants on each topic.

Additionally, the level of feedback received was examined. Three levels of feedback were established for participants’ responses: no feedback/low feedback (1 point), medium feedback (2 points), high feedback (3 points). Therefore, points were the result of an evaluation of the clarity of the answers in each topic. The overall level of feedback was also calculated per theme (Table 4), establishing low (13–21 points), medium (22–30 points), or high feedback (31–39 points).

Due to the cultural characteristics of participants, the dialogues were translated from Spanish to English. Quotes were used to illustrate the ideas expressed by the participants. “P” indicates participant, “MCI”, “DEM”, and “PR” (professional) the group type, and “G” the subgroup (e.g., P1(MCI)G1). Furthermore, for a better understanding of the contents, verbatim quotes were slightly modified.

It was impossible to receive the participant’s feedback on the transcription and findings as some participants could not be contacted for reasons beyond the researchers’ control. Results are explained per theme in the next section.

## 3. Results

Table 3 summarizes the positive and negative ideas collected in the focus group discussions and the most meaningful quotes registered. Table 4 shows the feedback (low, medium, high) and feedback scores

### 3.1. Usability (Learning and User-Friendliness)

GRADIOR 4.5 was considered intuitive and straightforward as it did not require previous experience with computers or other technologies. In the first few sessions, people with MCI and PWD received support from monitors, but immediately became familiar with the program. A minority of participants pointed out that mainly at the beginning, managing the computer program was not effortless. Furthermore, professionals remarked that the learning process might depend mostly on the user’s profile type, impairment degree, and previous experience with technological devices.

The therapist’s profile was valued as sophisticated and not intuitive. Some professionals found certain parts ambiguous, not evident, and confusing for people who do not frequently use the program.

The contributions of the MCI (30 points) and PWD (28 points) groups were highlighted in the user-friendliness category. The most precise response is the following quote concerning the ease of use of the tool: “Even if you don’t use the computer for a while, you know how it works. You don’t forget how to use it because it is easy” P8(MCI)G2. In the learning category, the MCI groups’ contributions were highlighted (27 points), and in general, the answers provided medium feedback.

### 3.2. User Experience (PWD/MCI and Therapist)

An aspect that participants with MCI and healthcare professionals highlighted was the positive experience using GRADIOR 4.5. Using the computer program was considered fun, entertaining, and a chance to learn and expand one’s social circle. The need to get to the workshop center was also valued as positive by PWD since it was considered a physically active method. Some participants also felt satisfaction when using computers, as they had no previous experience with technology.

Healthcare professionals perceived that PWD and MCI liked to use GRADIOR 4.5 and found it attractive and enjoyable. However, compared to other programs, GRADIOR 4.5 was not considered dynamic software. They also stated that engaging older adults with GRADIOR 4.5 was challenging, and the repeated use of the software was discouraging. Regarding the experience as a therapist, GRADIOR 4.5 was found enjoyable and useful for day-to-day work.

User experience was the topic with the most responses for the PWD (38 points) and MCI (33 points) groups. The answers were clear and relevant, as indicated by the following quotes: “At first, I didn’t like the computer at all. I didn’t think I was going to use a computer and get attached to it” P3(DEM)G2 and “The truth is that I don’t change the computer program for anything” P1(MCI)G1. Conversely, the feedback on the experience as a therapist was not abundant (14 points).

### 3.3. Acceptability (Perceived Usefulness, Duration of the Sessions, and Experience with GRADIOR 4.5)

The program was considered beneficial to learn, exercise memory, and recover lost skills. The MCI group commented that GRADIOR 4.5 mainly enhanced their memory and attention and helped manage daily life challenges. Besides, the PWD group considered the computer program an opportunity to spend time and get to know people of a similar age. Furthermore, according to professionals, older adults perceived GRADIOR 4.5 as part of a clinical treatment rather than a hobby or a game. Professionals underlined the program’s high acceptance in this population, even if some believed that the software benefits did not seem to be reflected in real life.

Regarding the sessions’ frequency and duration, people with MCI and PWD used GRADIOR 4.5 for half an hour twice a week. Participants were satisfied with the regular use rate, although they considered the sessions’ length too short. In terms of experience with the program, most participants with MCI and dementia used GRADIOR 4.5 for over 3–4 years. Half of the professionals had a long enough experience with the program, while the other half’s knowledge was a few months.

The contributions made by the PWD (32 points) and MCI (31 points) groups in the perceived usefulness category (MCI and PWD) were extraordinarily relevant and precise. The most specific answers were, “I come for the memory, I had a poor memory, I forgot things. I feel much better now; it is the truth. I have recovered memory” P2(DEM)G1 and “It came in handy for me; for the mind and memory, because I was not feeling very good” P4(MCI)G1. Regarding the session duration category, much information was also obtained in the MCI group (30 points). For the rest of the categories, no valuable feedback was obtained.

### 3.4. Accessibility (Technological Devices and Internet)

The program’s accessibility was considered low because older adults did not own a computer, nor did they have an Internet connection. Participants noted that the only way to use GRADIOR 4.5 was to go to the workshop center.

Accessibility was one of the categories with the least feedback obtained. Besides, the few responses on technological devices and the Internet subcategories were quite ambiguous.

### 3.5. Sustainability (Cost, Recommendation, and Using GRADIOR 4.5 Again)

In the MCI group, half of the users confessed that if the sessions with GRADIOR 4.5 had a price, they would stop attending the intervention. The other half agreed to pay something if the sessions contained other activities. Furthermore, GRADIOR 4.5 was considered to be recommended to family members, close friends, and neighbors as a way to spend free time, distract, exercise memory, and meet people of a similar age. For PWD, GRADIOR 4.5 was also excellent and beneficial to health. Participants in the PWD and MCI groups stated that they would like to use the computer program another time and were willing to try new technological programs to enhance memory. Moreover, according to the experience of the group of healthcare professionals, GRADIOR 4.5 was a useful tool for their work and highly recommendable to other therapists.

The contributions of the MCI (33 points) and PWD (35 points) groups provided clear answers in the recommendation to the user’s category. Among the most precise answers are the following quotes: “The truth is that it is highly recommended. At least you are exercising memory” P3(DEM)G1 or “It’s very nice to come, to share time together and remember things. I have recommended it to other people. At least you’re exercising your memory” P4(DEM)G1. In the rest of the categories (cost and using GRADIOR 4.5 again), low and medium feedback and ambiguous responses were collected.

### 3.6. Exercises (Speed, Difficulty Level, Instructions, Feedback, Variety, and Suggestions for Improvements)

Regarding the speed of activities, participants commented that the stimulus was displayed for a short time for some tasks. In general, professionals agreed with the ideas and considered modifying the stimuli’s speed and the response time. According to professionals, the instructions’ rate was adequate and enough to understand the exercises.

For most participants, the difficulty level of exercises varied according to the type of task. Specifically, one participant of the MCI group believed that some activities were too simple and time-wasting. However, the majority of the participants admitted being frequently confused even on the simplest tasks. According to professionals, specific exercises did not seem intuitive enough for older adults, which were necessary to support.

In general, the instructions were considered adequate, clear, and easy to understand. However, for some participants, the numbers of specific tasks were very few and not well noticeable. Similarly, healthcare professionals considered it unnecessary to provide both written and auditory instructions as older adults’ understanding may worsen. They also highlighted the benefits of giving feedback to older adults, and in general, the PWD and MCI groups confessed that receiving feedback messages made them feel good and happy. Furthermore, participants believed that the program contained various activities, even though the importance of incorporating a wide range of new exercises and difficulty levels was mentioned. Additionally, professionals suggested adding an example of the exercises and replacing the feedback messages with more positive expressions. They also agreed that the activities of the latest version of GRADIOR 4.5 had improved considerably in terms of image quality.

People with MCI (31 points) and healthcare professionals (29 points) provided much feedback on the topic’s difficulty level. The answers obtained were clear and relevant, as indicated by the following quotes: “I am very good at relating faces to names; where I have lost a lot is in the calculation, but someday I will do it right. Some exercises are difficult, but, in the end, you get it right” P2(MCI)G2; “For example, some exercise, especially executive function, users need explanations as they are not very clear, or they are not very clear to them” P3(PR)G2. In the instruction’s subcategory, reasonable responses were also collected, although answers were quite ambiguous. Moreover, the professionals’ answers were abundant in the topic suggestions for improvement (32 points).

### 3.7. User’s Profile (Hardware, Software, and Suggestions for Improvements)

Touch screens were considered the main advantage of the hardware. Professionals also felt that touchscreens made it easier for older adults to interact with the program and stay focused. Furthermore, participants considered the instructions and the size of the computers adequate. However, the screens’ low sensitivity and the necessity to use powerful equipment were considered significant disadvantages. As a hardware improvement suggestion, professionals highlighted the importance of using more sensitive touchscreens and adjusting screens to participants’ height to improve performance and comfort during the exercises. Besides, healthcare professionals suggested incorporating an intuitive function to regulate the volume and headsets with noise canceling.

The interface’s appearance was considered attractive, and the images, colors, and font sizes were appropriate. Professionals found the program’s interface engaging and emphasized that the most significant innovation was the stimuli, real images, and background color changes. According to professionals, one of the considerable disadvantages of the software was the accessibility for people who use the application at home without supervision. The pause button also was not considered intuitively located, and written explanations of some exercises seemed to overlap. Another significant disadvantage was the need to be connected to a high-speed Internet connection to run GRADIOR 4.5. As a suggestion for improvement, healthcare professionals proposed to adapt the content and make the software more dynamic. Furthermore, professionals missed facilities to search for users’ sessions on the main menu and proposed a function to advance exercises and add global feedback.

The PWD group’s contributions stood out in the advantages of the software category (27 points). The advantages of hardware were the topic with great feedback in the MCI group (30 points). Healthcare professionals stressed the disadvantages (30 points) and improvements of the software (31 points). Among the most precise explanations is the following quote: “The screen should be more sensitive, and the screen should be at the user’s height. I sometimes see people sitting very far and in uncomfortable positions to get to the screen” P2(PR)G2.

### 3.8. Therapist Profile (Hardware, Software, and Suggestions for Improvements)

Healthcare professionals agreed that the therapist interface was attractive and modern. One of the benefits highlighted was the easy access to the therapist’s profile, simplifying some steps, and the automation of some functions. As for disadvantages, the search for active treatments and the final reports were mentioned. Furthermore, the therapist’s profile was considered complicated and confusing. Suggestions included simplifying searching for active treatments, making treatment associations more intuitive, and adding explanations to some functions. Professionals also proposed the possibility of recommending or showing alternative exercises.

Many responses were collected in the software improvement category’s suggestions for the healthcare professionals’ group (31 points). Among the most precise explanations is the following quote: “I believe a mistake has been made, which is to put ‘treatments’ on the tab; ‘rehabilitation treatment’, ‘evaluation treatment’, and ‘baseline treatment’. The treatment is the treatment, the evaluation is the evaluation, and the baseline is the baseline. They are not all treatments. The word ‘treatment’ should disappear” P5(PR)G1. The feedback on the software advantages (22 points) and disadvantages (24 points) was not abundant, and medium feedback was collected.

### 3.9. Comparison with Other Programs (Preferences, Type of Activities, and Suggestions)

During the discussions, participants in the PWD and MCI groups were asked about their computerized software preferences and other traditional activities. Half of the participants favored traditional interventions, while the other half chose computerized ones. The short duration and characteristics of the GRADIOR 4.5 sessions (individualized) were mentioned as weaknesses, despite requiring a lower cognitive level than traditional activities. Similarly, according to professionals, one of the most significant advantages of GRADIOR 4.5 was the support it provided for their work. Professionals also suggested incorporating cognitive stimulation video games to make GRADIOR 4.5 more dynamic and attractive. Mainly, getting older adults engaged was found challenging as the exercises were considered too clinical. Another idea was to incorporate reminiscence exercises in the software.

Preferences for the type of intervention was the topic with the most responses. However, the participant’s answers were mostly ambiguous and provided low and medium feedback. Similarly, the feedback on the type of activity and suggestion categories was not abundant.

## 4. Discussion

The study presents the outcomes of a qualitative study using the focus group methodology, in which the cognitive rehabilitation software GRADIOR 4.5 is evaluated. Potential users with MCI and dementia and healthcare professionals were selected to assess usability, user experience, acceptability, accessibility, sustainability, and other aspects related to computerized programs.

### 4.1. Learning and User-Friendliness

Usability is one of the essential requirements that software must satisfy [33]. In this study, usability was measured by the ease of learning and user-friendliness of the tool. The results indicated that learning to use GRADIOR 4.5 was considered simple and that its use was quite intuitive, especially for older adults with cognitive impairment. However, differences were found between the PWD and MCI groups regarding the facility to handle the software autonomously and learning to use it. These difficulties are to be expected due to the characteristics of the target group, which, in this case, were older adults with cognitive impairment and without much previous experience with digital devices and software. Therefore, people with MCI and dementia will need training until they are able to handle the software autonomously. These results are consistent with a previous study where the efficacy of an integrated technology platform that combines cognitive exercises with physical activity was assessed [20].

### 4.2. Users’ Experience

Some systems are designed to be used autonomously by users; however, using auto-run computer programs may be challenging for people with cognitive impairment. Consequently, we support the need to apply these systems with the therapist’s help (at least in the beginning) to guarantee fair use and engagement. Indeed, people using GRADIOR 4.5 for the first time are always supported until they become familiar with the tool. According to healthcare professionals participating in this study, it is estimated that older adults with MCI or dementia need 3–4 sessions to get used to GRADIOR 4.5. These findings are similar to other studies. Healthy older adults with no previous experience with technologies found it easy to manage a technological tool and felt comfortable with it in the first week [34].

### 4.3. Acceptability

Acceptability is another necessary condition for the successful implementation of computerized cognitive programs. This characteristic also increases the clinical benefits of interventions [35]. The present study’s findings suggest that people with MCI and dementia widely accepted GRADIOR 4.5 as its use was linked to memory and attention enhancement, recovery of lost skills, and better management of daily life activities. These results are consistent with another study. The degree of acceptance of an Information and Communications Technology (ICT) platform to promote health, independence, and quality of life in older adults with MCI was measured [36]. In our study, a further indicator of acceptability was the gathered information regarding the sessions’ frequency of use and duration. Participants with MCI and dementia expressed dissatisfaction with the workshop’s length (where they used GRADIOR 4.5), which leads us to believe that they were hugely motivated and engaged in the program. However, some healthcare professionals questioned the usefulness of the GRADIOR 4.5 software as in other effectiveness studies [37,38]. Thus, a clinical trial will be conducted to analyze the GRADIOR 4.5 program’s effectiveness compared to a psychosocial intervention program [27].

On the other hand, one of the advantages of computerized programs is that they are dynamic, motivating, and entertaining [39]. In this study, participants showed high satisfaction with the software. Indeed, GRADIOR 4.5 appeared to imply having fun, learning, and staying active, which suggested that it was a positive experience for people with MCI and dementia. Another indicator of the positive experience was the recurring thought of having the opportunity to meet people. This is an essential point because one of the most frequent computer program criticisms is the isolation and difficulties of promoting social networks among older adults. However, it seems beneficial to use these systems in shared rooms to interact with others. These results are in line with those obtained in the study by Contreras-Somoza et al. [36].

### 4.4. Accessibility

The results obtained concerning the accessibility of the software were not surprising. GRADIOR 4.5 requires a robust Internet connection in addition to computer equipment that, considering the characteristics of the study participants, we can anticipate most people with MCI and dementia do not own. Therefore, participants in this study had to travel to a center where the necessary equipment was available. These results are supported by previous studies that identified Internet availability as one of the significant barriers to engage older adults in the use of technology [12]. However, going to the center can promote social relationships and physical activity, as some participants mentioned.

### 4.5. Sustainability

GRADIOR 4.5’s sustainability results were also as expected. When people with MCI and dementia were asked about the possibility of paying for GRADIOR 4.5, most people did not feel comfortable. Older adults may have low monthly incomes, so it is reasonable to consider that paying for a computer program is an economic burden that they probably could not afford. These results are supported by studies where the price was identified as a barrier for older adults to use technology [12]. However, according to a recent study, the use of technology in older adults would be more influenced by a lack of confidence in handling technology than by the devices’ price [13]. Interestingly, in our study, some participants were willing to pay a small quantity of money for the computer program, which suggests that they might value the perceived benefits above the cost of technology [40].

### 4.6. Exercises

Concerning GRADIOR 4.5’s exercises, participants mentioned that some activities were far more complicated than others. We considered it a positive aspect that users thought that some tasks were demanding. GRADIOR 4.5 aims to rehabilitate cognitive functions such as memory and attention. Therefore, exercises must be continuously challenging for the recovery of cognitive skills. When faced with easy tasks, users may get bored and stop being engaged, and on the contrary, if all the exercises were of a high level, users may become frustrated and discouraged. Finding a balance between the levels of difficulty and the user’s capabilities would intervene with success. Indeed, it is fundamental that rehabilitation software contain different levels of difficulty so that the exercises can be adjusted to each user’s profile and needs. Considering that no cognitive rehabilitation program is designed exclusively for one type of population or pathology [14], it is predictable that all users will not perform the same on the tasks. The positive aspect of this type of program is that by containing so many difficulty levels and quantitative data related to their performance, treatment can be correctly adjusted.

### 4.7. Users’ Profile

Participants agreed that computers with a touchscreen were the simplest way to run the user interface. These results are consistent with other usability studies on tablet computers for people with early-stage dementia [8]. Furthermore, considering the research results, reviewing participants’ proposals to strengthen the program is recommended. In short, the study participants proposed to avoid interference between auditory and written instructions, prevent overlaps between instructions and parts of the exercises, increase their number, place stimuli and the pause button in more intuitive locations, facilitate the search for user sessions, replace feedback messages with more positive ones, and simplify the accessibility to the program. Participants also suggested incorporating new functions such as global feedback at the end of the sessions, new exercises for each cognitive subdomain, more exercise levels, examples before each task, an intuitive function to regulate the volume, the ability to advance exercises, stimulating video games, and reminiscence exercises.

### 4.8. Therapist Profile

Furthermore, the therapist’s area was considered sophisticated and required continuous use to maintain familiarity. However, healthcare professionals indicated that they found it beneficial to use this tool as part of their work and considered GRADIOR 4.5 a highly recommended support for other healthcare professionals. Tools such as GRADIOR 4.5 offer significant advantages for professionals, such as providing support to clinical work, saving time in the analysis of exercise performance, and not requiring highly qualified training. Consequently, it is easier to develop and deploy a systematized computerized rehabilitation tool instead of the usual interventions in many care centers. However, in this study, healthcare professionals suggested improving the search for active treatments and the association of treatments and explaining some functions and recommendations of alternative exercises. These suggestions should be considered to improve the handling of and experience with GRADIOR 4.5 as a therapist.

### 4.9. Comparison with Other Programs

Different opinions were obtained regarding preferences between traditional and computerized activities. Participants who chose conventional programs argued that the workshops were usually longer and that the exercises were done in groups, which allowed them to spend more time enjoying and interacting with other participants. However, many other participants agreed that they were no longer able to perform group activities since traditional interventions require more writing skills. Participants also recognized that the computer program was better suited to their current cognitive capabilities.

### 4.10. Level of Feedback Received

Additionally, our study analyzed the degree of communication or feedback collected in each category. The group of professionals provided more information in the categories related to aspects to be improved. In contrast, user experience, perceived usefulness, and recommending GRADIOR 4.5 to other users were the most prominent categories for the MCI and PWD groups.

Differences in group contributions were estimated, taking into account the characteristics of the groups of participants. The study considered the healthcare professional’s view. They understood and used it from another perspective and were aware of more technical aspects that could be interesting for a successful computerized program intervention. Therefore, it is reasonable to think that professionals would be more critical of the weaknesses of GRADIOR 4.5 and what could be improved. For instance, the healthcare professionals proposed to include new functions to regulate the volume, advance exercises, and provide general feedback. They also saw the development of dynamic exercises, creating more difficulty levels, and providing positive feedback messages necessary. They also proposed to simplify some functions and add explanations in the therapist profile.

An earlier version of GRADIOR was part of the Long Last Memories program (LLM) that combines cognitive exercise and physical activity in an integrative platform [24]. The previous usability study evaluated aspects such as ease of use, sustainability, and satisfaction from Spanish older adults [19,26]. The study’s findings suggest that the LLM platform was easy to learn and use, highly recommended, and well accepted by older adults. Therefore, the results of our research are supported by the previous usability study of GRADIOR.

### 4.11. Limitations and Suggestions for Future Research

Some limitations should be considered. First, the sample was selected to be representative and to cover different points of view. Even though it was not deliberate, the participants were predominantly women. Secondly, the MCI and dementia groups had only had experience with GRADIOR 4.5 software, so they could not compare it to other programs. Anyway, the aim was not to compare different software, but to know the main features for considering the implementation and acceptability of cognitive training computer-based programs in clinical settings to deliver this treatment inexpensively and affordably. Furthermore, information on participants’ previous experience with technological devices such as tablets or smartphones was not collected. Another weakness of the study was that not all types of responses were included in the analysis (e.g., non-verbal responses), so we may have missed valuable information. The healthcare professionals participating in the study were INTRAS Foundation employees, the prime promoter of GRADIOR 4.5 software. However, professionals’ opinions and comments were not influenced since there was no interaction between the clinical professionals and the software developers. Indeed, the group of professionals evaluated GRADIOR 4.5 quite critically in comparison with other study participants.

On the other hand, the focus group methodology may not be the most suitable technique for people with MCI and PWD, as discussions in the MCI group frequently diverged from the study subject, and the PWD group mainly provided yes and no answers. As might be expected, PWD’s participation was a little lower than other groups. It was reflected in the analysis of the degree of feedback and the duration of the focus groups. Besides, considering that participants in the PWD and MCI groups may experience memory loss, participants may not have reflected everything they thought about GRADIOR. Perhaps, it would have been convenient to use supporting material such as videos, screenshots, or photos of the computer program’s contents. It is, therefore, necessary to take different approaches on the same subject, considering all the various parties involved, such as patients and professionals, and by methods such as focus groups or even questionnaires [25]. However, the researchers took the inclusive participation of PWD in their study very seriously and tried to the best of their knowledge to overcome challenges and address the needs of PWD to achieve this.

Lastly, we would like to emphasize the importance of conducting usability studies in the cognitive rehabilitation software field. Several computer programs had been identified from related published studies [41]. Most studies focus on the effectiveness or benefits of computer-based cognitive tools rather than on usability and acceptability aspects that could influence the implementation of computerized interventions [14]. Future research could be focused on assessing the usability of technologies to understand better the potential barriers related to computerized cognitive rehabilitation. Further studies are also needed to promote the social inclusion of people with dementia in the process of the design and evaluation of care solutions.

## 5. Conclusions

In this study, the cognitive rehabilitation software GRADIOR 4.5 was evaluated to determine whether it is an adequate tool for people with MCI, mild dementia, and healthcare professionals. The findings showed that technologies could be entirely employed as part of a cognitive rehabilitation intervention for people with MCI and dementia, mainly due to its high acceptability and sustainability. The study also indicated a positive attitude towards technology by older adults. People with MCI and dementia requested to use the program longer, which might mean enjoyment and firm adherence to treatment. In general, paying for this type of service is not common because treatments are typically not paid for in Spain. In any case, the viability of incorporating these treatments into the system, in terms of sustainability, should be assessed. Healthcare professionals detected other limitations and resistance to the program. Hence, the professionals highlighted the need to improve the program in terms of accessibility and increase the program’s dynamics by incorporating new exercises. This means that probably the main barriers to implementing this kind of methodology come from the professionals and change resistance. Lastly, although the study’s results and recommendations refer specifically to the GRADIOR 4.5 rehabilitation program, it would be advisable to consider these findings in future developments. Furthermore, we understand that this type of system needs a systematized implementation in the cognitive intervention area due to the opportunities offered. It will be necessary to improve the professionals’ training and make affordable the application of this approach. The best is to offer a comprehensive service of cognitive training or psychosocial intervention at home and day centers.

## Figures and Tables

**Table 1:** Sociodemographic characteristic of study participants.

Participants	Gender	Age	Marital Status	Educational Level	Time Using GRADIOR
MCI					
P1(MCI)G1	Female	83	Widowed	Read and write	3 years
P2(MCI)G1	Male	59	Unmarried	Secondary school	3 months
P3(MCI)G1	Female	76	Married	Secondary school	4 years
P4(MCI)G1	Female	77	Widowed	Secondary school	4 years
P5(MCI)G1	Male	69	Married	Primary school	4 years
P1(MCI)G2	Female	71	Separated	Secondary school	7 weeks
P2(MCI)G2	Female	82	Widowed	Primary school	2 years
P3(MCI)G2	Female	82	Divorced	Primary school	5 years
P4(MCI)G2	Female	80	Widowed	Read and write	2 years
P5(MCI)G2	Female	65	Widowed	Primary school	3 years
P6(MCI)G2	Female	78	Married	Primary school	6 weeks
P7(MCI)G2	Female	87	Widowed	Primary school	3 years
P8(MCI)G2	Female	70	Widowed	Primary school	3 years
PWD					
P1(DEM)G1	Male	86	Married	Read and write	2 months
P2(DEM)G1	Female	79	Widowed	Secondary school	1 month
P3(DEM)G1	Female	71	Separated	Primary school	6 months
P4(DEM)G1	Female	78	Widowed	Read and write	3 years
P5(DEM)G1	Female	69	Widowed	Primary school	4 years
P6(DEM)G1	Female	80	Widowed	Read and write	6 weeks
P1(DEM)G2	Male	93	Widowed	Primary school	4 years
P2(DEM)G2	Female	77	Widowed	Read and write	3 years
P3(DEM)G2	Female	72	Married	Secondary school	7 months
P4(DEM)G2	Female	50	Separated	University degree	3 years
P5(DEM)G2	Female	77	Widowed	Primary school	8 months
P6(DEM)G2	Male	73	Unmarried	Primary school	1 months
P7(DEM)G2	Male	87	Unmarried	Primary school	1 year
Healthcare professionals					
P1(PR)G1	Male	50	-	-	7 years
P2(PR)G1	Female	33	-	-	6 years
P3(PR)G1	Female	29	-	-	7 years
P4(PR)G1	Female	38	-	-	13 years
P5(PR)G1	Female	38	-	-	13 years
P6(PR)G1	Female	27	-	-	1 year
P1(PR)G2	Female	24	-	-	2 months
P2(PR)G2	Male	26	-	-	6 months
P3(PR)G2	Male	39	-	-	3 months
P4(PR)G2	Female	35	-	-	9 years
P5(PR)G2	Female	25	-	-	3 months

G = group; DEM = dementia; MCI = mild cognitive impairment; PWD = people with dementia; PR = professional.

**Table 2:** Semi-structured and open-ended questions used in focus group discussions.

**MCI and PWD Groups’ Script**
1. What do you think about the program? 2. Do you like using it? 3. How often do you use it? 4. Do you find it easy to use? a. What was the hardest thing to learn about the program? 5. Do you think that the instructions given by the computer are clear and easy to understand? a. Do you hear the instructions loud and clear? b. The letters and numbers that appear on the screen are the right size? 6. Do you feel that GRADIOR can be of benefit to you? 7. What do you like most about the program? (advantages) 8. What do you like least about it? (disadvantages) 9. How do you like the overall look and feel of the program? 10. Do you think that the touch system (touching the screen with your finger) makes it easier for you to use GRADIOR? 11. Would you like to use GRADIOR again if you had the chance? 12. Would you like your family, friends, neighbors, etc. to use the program and recommend it to them?
**Healthcare professional groups script**
1. From your experience with the old version and the new version of GRADIOR, what do you think has been the biggest improvement or innovation in the program? a. Are there any aspects that have worsened? 2. What are the advantages of the new GRADIOR compared to previous versions? 3. What are the disadvantages of the new GRADIOR compared to previous versions? 4. Do you think that the new version of the program is sufficiently adapted to your users (taking into account their characteristics and needs)? 5. What do you think the appearance of the program could be improved? 6. About the therapist’s profile, what functions would you add to this profile? 7. When creating the list of exercises or the treatment for each user, how do you think the system works? a. Do you think that it could be improved to make it more intuitive, simpler, etc.? 8. Regarding the reports obtained with the GRADIOR program, do you think that there is any aspect that could be improved in the collection, handling and interpretation of the data? 9. Have you encountered any technical problems that limit the overall performance of the program? a. And for the users? 10. From your experience, do you find that users like using GRADIOR? 11. Do you think it was difficult for users to use GRADIOR? a. Do they need any kind of help to use it normally? 12. What do you think about the overall look and feel of GRADIOR? 13. Do you think that the instructions given by the computer are clear and understandable to the users (audio instructions)? a. What about the written instructions on the screen? 14. What do you think about the size and characteristics of the computers on which GRADIOR is used (size and touch screen)? 15. Do you think that the speed at which the exercises are presented is adequate for the users? 16. What do you think about the feedback that users receive after each exercise (the program tells them if the answer they gave is correct, incorrect or if they have missed the time)? 17. Would you recommend the GRADIOR program to other professionals?

**Table 3:** Overview of positive and negative ideas expressed by participants in the focus groups.

Theme	Positive ideas	Negative ideas	Quotes
Usability	Simple Intuitive	At first difficult Sophisticated (TP) Ambiguous and confusing (TP)	P4(MCI)G1: *“Not at first, but now I find it easy to use.”* P3(PR)G1: *“It’s very intuitive, it’s very easy to use for those people with cognitive impairment or not, and for people who have experience with computers or not.”* P6(PR)G1: *“The therapist area is not so intuitive and easy to learn for me; I still get lost.” (TP)*
User’s experience	Positive experience Entertaining, enjoyable Attractive Expand social circle Enjoyable (TP)	Not dynamic	P2(MCI)G2: *“I love it. I am always thinking ‘Oh, tomorrow I have to go, I cannot forget’, ‘Oh, I have to go today’. I like the whole computer. I feel happy, I have never used a computer in my life.”* P5(DEM)G1: *“I like the computer, but I’m not good. I come for going out of house and interacting with people.”* P3(PR)G1: *“The truth is that it is more pleasant, we feel happier.”*
Acceptability	Learn, exercise memory, and recover lost skills Spend time Meet new people Improve cognition	Benefits not reflected in real life Too short sessions	P5(DEM)G2: *“It makes you think. For example, are they chickens or cows? You have to be focused, and you have to look at those that look to the left, those that look to the right…”.* P1(MCI)G1: *“It’s very short. It certainly doesn’t even give us time to sit down, because with half an hour. I want to do a little more.”* P3(PR)G2: *“In the end, what brings you in real life? You do not see an improvement in attention or memory or general skills.”*
Accessibility	−	Low accessibility Powerful computer Internet connection	−
Sustainability	Advisable Healthy Distractive Useful (TP) Highly recommendable (TP)	Need to pay	P2(MCI)G2: *“It is good for you; it distracts you; your mind is engaged in something positive”.* P3(DCL)G1: *“I’m not going if I have to pay”.*
**Theme**	**Positive ideas**	**Negative ideas**	**Quotes**
Exercises	Adequate rate of instructions Understandable instructions Receiving feedback Wide range of exercises The image quality of exercises	Short display of the stimulus Low response time Basic activities Non-intuitive exercises Small number size Undeveloped modalities Lack of difficulty levels Feedback messages	P8(MCI)G2: *“When they put the instructions “catch a bird” and when you look there’s no more”.* P3(PR)G1: *“I believe that the stimuli have been changed, but no new exercises have been generated. Drawings had change for real photos, but in fact, the same exercises are going to be done all the time. We need new exercises”.* P3(MCI)G1 *“I think some exercises are very elementary. The program always asks us what day it is and in what season we are. I think this takes time away from other exercises because we all know in what year and season we are”.*
User interface	Touch screen Adequate size of computers Attractive appearance Appropriate images, colors, and font size	Low screen sensitivity Powerful equipment Accessibility from home Search for users’ session Non-intuitive stimulus	P1(DEM)G1: *“It seems to be easier to use the computer with the finger than with the keyboard”.* P2(PR)G1: *“It is a real image that favors the later recognition, because sometimes with a predesigned image someone with cognitive impairment that does not have mental retention that is, I do not know, can be something else”.*
Therapist interface	Attractive and modern Adaptation of the contents Access to therapist’s area Simplification of steps Automation of functions	Search for active treatments Final reports data Complicated interface	P3(PR)G2: *“For example, the baseline is done automatically and in the previous version it was done manually. It is the change that I see. Then there has been a favorable progression”.* P5(PR)G1: *“When we do reviews of the treatments there was an option to cancel the previous treatments and keep in the viewer only the current ones. Now are all the treatments of all current people. Maybe you have 200 treatments and it’s awfulness to look for the treatments you want to find”.*
Comparison with other programs	Therapist’s support	Not dynamic Not attractive Get older adults attached	P8(DCL)G2: *The only thing is that in GRADIOR you can’t talk at all. In the pencil and paper workshop, you have more choice to give your opinion. But I am very happy with both activities”.* P2(PR)G2: *“The exercises are fine, but many times they are very de-contextualized, very neuropsychological. Doing them as a video game, I think maybe it would make it a little more attractive, although it’s complicated”.*

TP = therapist’s profile.

**Table 4:** Level of feedback reached by groups and categories.

Themes	MCI	Dementia	Professionals
Usability			
Learning user’s area	Medium (27)	Low (21)	Low (23)
Learning therapist area	NA	NA	Low (16)
User-friendliness user’s area	Medium (30)	Medium (28)	Medium (22)
User-friendliness therapist area	NA	NA	Low (17)
User experience			
PWD and MCI	High (33)	High (38)	Medium (26)
Therapist	NA	NA	Low (14)
Acceptability			
Perceived usefulness by MCI and PWD	High (31)	High (32)	Medium (14)
Perceived usefulness by therapist	NA	NA	Low (20)
Duration of the sessions	Medium (30)	Low (19)	Low (11)
Experience with GRADIOR 4.5	Low (15)	Low (17)	Low (11)
Accessibility			
Technological devices	Low (14)	Low (20)	Low (12)
Internet	Low (14)	Low (13)	Low (11)
Sustainability			
Cost	Low (18)	Low (13)	Low (11)
Recommendation to users	High (33)	High (35)	Medium (22)
Using GRADIOR 4.5 again	Medium (24)	Medium (26)	Low (11)
Exercises			
Stimulus speed	Low (16)	Low (15)	Low (15)
Response time speed	Low (20)	Low (19)	Medium (22)
Instruction speed	Low (13)	Low (13)	Low (16)
Difficulty level	High (31)	Medium (24)	Medium (29)
Instructions	Medium (24)	Medium (26)	Medium (23)
Auditory instructions	Medium (23)	Low (16)	Low (11)
Written instructions	Medium (22)	Medium (25)	Low (18)
Feedback	Low (18)	Medium (24)	Medium (22)
Variety of exercises	Low (17)	Low (13)	Low (18)
Suggestions for improvements	Low (15)	Low (17)	High (32)
User’s interface			
Hardware advantage	Medium (30)	Low (21)	Low (14)
Hardware disadvantage	Low (14)	Low (17)	Medium (23)
Suggestions for hardware improvement	Low (13)	Low (13)	Low (20)
Software advantage	Low (17)	Medium (27)	Medium (26)
Software disadvantage	Low (19)	Low (16)	Low (30)
Suggestions for software improvement	Low (13)	Low (13)	High (31)
Professional’s interface			
Hardware advantage	NA	NA	Low (11)
Hardware disadvantage	NA	NA	Low (11)
Suggestions for hardware improvement	NA	NA	Low (11)
Software advantage	NA	NA	Medium (22)
Software disadvantage	NA	NA	Medium (24)
Suggestions for software improvements	NA	NA	High (31)
Comparison with other programs			
Preferences	Medium (25)	Medium (24)	Low (21)
Type of activities	Low (21)	Low (19)	Low (16)
Suggestions	Low (13)	Low (13)	Low (21)

NA *=* not applicable.

## Data Availability

Data sharing is not applicable to this article.
